# Evidence-informed decision about (de-)implementing return-to-work coordination to reduce sick leave: a case study

**DOI:** 10.1186/s12961-022-00823-4

**Published:** 2022-02-14

**Authors:** Christina Tikka, Jos Verbeek, Jan L. Hoving, Regina Kunz

**Affiliations:** 1 Finnish Institute of Occupational Health, Neulaniementie 4, 70101 Kuopio, Finland; 2 Department of Public and Occupational Health, Amsterdam UMC, Location AMC, University of Amsterdam, Coronel Institute of Occupational Health, Cochrane Work Review Group, Amsterdam, North Holland The Netherlands; 3 Research Unit Evidence-based Insurance Medicine, Department of Clinical Research, University of Basel and University Hospital Basel, Basel, Switzerland

**Keywords:** Sick leave, Work ability, Sickness absence, Occupational health services, Occupational health policy, Evidence-based, Return to work

## Abstract

**Background:**

Coordination of return to work (RtW) is implemented in many countries, but a Cochrane review found no considerable effect on workers’ sick leave compared to usual care. The aim of the study is to analyse how the evidence from this review can be used for decisions about (de-)implementing RtW coordination in a country-specific setting, using Finland as an example.

**Methods:**

We conducted a systematic literature search and online survey with two groups of experts to compare interventions included in the Cochrane review to Finnish RtW practice using content analysis methods. We applied the evidence-to-decision (EtD) framework criteria to draw conclusions about (de-)implementing RtW coordination in Finland, including benefits, harms and costs of the intervention compared to usual care.

**Results:**

We included seven documents from the literature search and received data from 10 of 42 survey participants. RtW coordination included, both in Finland and in the review, at least one face-to-face meeting between the physician and the worker, a workers’ needs assessment, and an individual RtW plan and its implementation. Usual care focuses on medical treatment and may include general RtW advice. RtW coordination would be cost-saving if it decreases sick leave with at least 2 days compared to usual care. The evidence in the Cochrane review was mainly of low certainty, and the effect sizes had relatively wide confidence intervals. Only a new, high-quality and large randomized controlled trial (RCT) can decrease the current uncertainty, but this is unlikely to happen. The EtD framework did not provide arguments for further implementation or for de-implementation of the intervention.

**Conclusions:**

Interventions evaluated in the Cochrane review are similar to RtW coordination and usual care interventions in Finland. Considering all EtD framework criteria, including certainty of the evidence and costs, de-implementation of RtW coordination interventions in Finland seems unnecessary. Better evidence about the costs and stakeholders’ values regarding RtW coordination is needed to improve decision-making.

**Supplementary Information:**

The online version contains supplementary material available at 10.1186/s12961-022-00823-4.

## Background

In many countries, sickness absence from work has since long been recognized as a public health problem with serious negative effects on workers and their families, as well as on employers, government and the society [1, 2]. In Europe, average absence rates range between 3 and 6% of working time with costs estimated at 2.5% GDP [3]. Lack of coordination in the return-to-work (RtW) process is often thought to be the cause for prolonged sick leave [4, 5]. Thus, improving communication and collaboration between employer, employee and occupational health service providers is a common approach in many countries with the aim to decrease sick leave. However, evidence from a Cochrane review could not show a different effect of RtW coordination on the duration of sick leave compared to usual care [6].

While the Cochrane review includes the best available evidence and found RtW coordination and usual care similarly effective, it is yet unclear how the evidence can be translated to RtW practice and policies. Especially when studies do not find beneficial effects of an intervention, practice and setting can be perceived differently from what is evaluated in these studies, and results are not translated to practice [7]. For example, in Finland, while the Cochrane review could not find a beneficial effect of RtW coordination compared to usual care [6], the Finnish government considerably invested in the development and implementation of RtW coordination [8, 9]. Ideally, de-implementation studies can show us when to stop investments and distribution of interventions that have been proven inefficient or have been overtaken by more efficient intervention [10, 11]. Yet, evidence-informed health system and public health recommendations and decisions should consider many factors. According to the Grading of Recommendations Assessment, Development and Evaluation (GRADE) evidence-to-decision (EtD) framework, those include the effect of the intervention, how substantial the benefits and harms are, the certainty of the evidence, how much people value the main outcomes, and the cost-effectiveness [12]. The main aim of the framework is to support transparent healthcare decisions that are informed by the best available research evidence and consider the wider social and political environment. While the framework has been applied to a variety of different health system and public health decisions, it has not yet been used for decisions regarding (de-)implementing RtW coordination interventions. We used Finland as an example to analyse the disparity between the Cochrane review evidence and RtW practice and to illustrate an example for making evidence-informed decisions regarding coordinating RtW.

## Methods

The aim of the study is to (i) analyse how comparable the interventions in the Cochrane review [6] are to RtW coordination practice in Finland and (ii) draw conclusions on (de-)implementing RtW coordination in Finland using the GRADE EtD framework [12].

### Data collection

We conducted a systematic literature search in MEDLINE using PubMed, webpages of Finnish research and government institutes, and reference lists of included studies (Additional file 1: Table S3) for any type of publication describing or evaluating interventions to coordinate RtW in Finland.

We updated the original Cochrane review literature search for MEDLINE using PubMed [6] until 7 March 2019 to judge whether new studies were available that fulfilled the inclusion criteria and were likely to change the results of the review. One researcher (CT) screened titles and abstracts for eligibility.

We further developed an online survey (Survey Monkey) to collect data about the difference between RtW coordination and usual care for workers on sick leave in Finland (Additional file 1: Table S3). We invited experts on RtW coordination in Finland to participate in the survey if they were (i) researchers who participated in the development of a training course on RtW coordination in Finland [13] or (ii) training course participants. Course participants and developers were invited to the survey in March and April 2019 via email, either through the course coordinator or directly (CT). Reminders were sent after 1 and 2 weeks.

### Comparison of interventions

We used content analysis methods to extract and summarize the literature and survey data. Prior to data extraction, we defined categories (such as content of RtW coordination) and corresponding themes (meetings, workers’ needs assessment, RtW plan, implementation management) based on the description of interventions included in the review (Additional file 1: Table S1). One author (CT) collected data for each category (names, year, setting, participants, content) for RtW coordination and usual care interventions in the review and Finland using Excel. Data analysis was done independently by two authors (CT and JV). Within each category, authors summarized similarities and differences between RtW coordination in Finland and the review and judged each category as identical (no differences), similar (more similarities than differences), different (no similarities) or unclear (missing data). Judgements were compared using Excel, and disagreements resolved via discussion.

### Using the EtD framework

The EtD framework for health system and public health recommendations [12] is structured into three parts: formulating the problem, making an evidence-informed assessment, and drawing conclusions.

#### Formulating the question

We formulated the question, including the interventions (RtW coordination versus usual care), the main outcomes (length of sick leave and number of workers returning to work), the setting (Finland) and the perspective from which the decision is being made (population perspective).

#### Making an evidence-informed assessment

For each framework criteria (Table 2), we summarized the available evidence from the Cochrane review, the systematic literature search and the survey. Due to missing cost-effectiveness studies, we used the best available data to analyse the costs of coordinated RtW interventions compared to usual care in Finland (Additional file 1: Table S2). We took the population perspective and considered costs and savings within the healthcare system. We compared the costs of sick leave for all workers on long-term sick leave in Finland with RtW coordination to those without RtW coordination. We used the effect of RtW coordination on sick leave to calculate the effect that this would have on the costs of sick leave.

#### Drawing conclusions

After the assessment, we drew our conclusion in relation to (de-)implementing RtW coordination in Finland. We formulated a summary of the most important judgements that were influencing our decision.

## Results

### Data collection

We included six publications that described the content and process of RtW coordination interventions in Finland. Publications were either recommendations and part of information and training material [9, 14, 15] or empirical studies conducted between 2014 and 2018 [16–18]. The update of the Cochrane review literature search identified 2858 references, including duplicates. Three studies with 98–180 participants were eligible for inclusion in the review [19–21]. Participants were workers on sick leave due to neck or shoulder pain, common mental disorders or injuries. We invited 39 of 42 eligible survey participants due to missing contact information. Two participants declined participation, and we received responses from 24% (10 of 42).

### Comparison of interventions

We judged RtW coordination and usual care interventions in Finland and those evaluated in the Cochrane review [6] to be mostly similar (Table 1). Coordination of RtW included at least one face-to-face meeting between the physician and the worker, who was often joined by the employer. In these meetings, participants discussed the progress of RtW and temporary work accommodations. The workers’ needs assessment consisted of an evaluation of the workers’ disability and functioning as well as considering factors from the type of work and the workplace. The RtW plan contained goals, such as full or partial RtW or being available for the labour market in other ways, and multiple actions, such as temporary work accommodations, medical interventions or psychological therapy. The plan was jointly developed by the healthcare professionals and the worker, but other participants could also join the development process, such as the employer or the worker’s support person. Mostly, the employer or the occupational physician was responsible for implementing the RtW plan and contacting the worker to ensure goals were achieved. The plan could be changed if this were deemed appropriate. In the Cochrane review, interventions always included a workers’ needs assessment that focuses on workability and barriers for RtW and an individually tailored RtW plan. According to Finnish recommendations, this is very similar to interventions in Finland. However, in practice, not all components of the RtW coordination intervention are delivered as intended. Low adherence to the study intervention was described for two out of the 14 included trials in the review. Similarly, the survey results showed that in Finland, the content of the workers’ needs assessment and the RtW plan does not always comply with the recommendations and might only include factors that are either related to the individual worker or his workplace (Additional file 1: Table S5).

**Table 1:** Similarities and differences between RtW coordination interventions in the Cochrane review and in Finland

Categories	Summary	Judgement
Names	Mostly use of keywords that suggest coordination, only some studies in the review used keywords that did not suggest coordination (such as case management and consultation)	Identical
Setting	Interventions are mostly situated in European welfare states and can start after a long-term sick leave of the worker In the Cochrane review all workers were at least 4 weeks on sick leave, but almost half of the Finnish workers had less than 4 weeks accumulated sick leave and may not have been on sick leave at the time of the RtW meeting	Similar
Year(s) studied	Data from different but overlapping time spans^a^, most studies were recent and conducted after the year 2000	Similar
Participants	The worker, the employer or a workplace representative, and a physician (most often occupational physician) participate in the intervention. Possibility to participate in the intervention for other healthcare providers (such as occupational health nurse or physiotherapist) and stakeholders (such as occupational safety representative, social worker)	Identical
Content	No differences: Interventions include: At least one face-to-face meeting between worker and coordinator, which is often but not always joined by the employer A workers’ needs assessment that includes a focus on employee's work ability A collaboratively developed RtW plan which consists of dates, goals and actions for RtW One person responsible for the implementation of the RtW plan (evaluating the progress and making changes to the RtW plan if appropriate) In practice, the RtW coordination intervention might not always be fully implemented as recommended	Identical
Duration^b^	In the review, interventions lasted 3 months until more than 1 year. Information about the duration of the intervention in Finland was missing	Unclear

^a^Review data: 18 years (1995–2016). Finland data: 5 years (2014 and 2018)

^b^Defined as from first meeting until last follow-up

In both the Cochrane review and Finland, the main differences between RtW coordination and usual care were that usual care mostly focuses on medical treatment, does not provide an individual tailored RtW plan and does not include considerations of the work or the workplace (Additional file 1: Tables S5 and S6). Also, in usual care the worker may receive general advice to return to work, and communication between healthcare providers is possible.

### Using the EtD framework

#### Formulating the problem

From the population perspective, should coordinated RtW be used in Finland to reduce the length of sick leave and the number of workers returning to work?

#### Making an evidence-informed assessment

We made judgements for all assessment criteria specified in the framework (Table 2).

**Table 2:** Assessment criteria and judgements for RtW coordination in Finland

Criteria	Judgement
Priority of the problem	Long-term sick leave has an important impact on the worker and the society in terms of productivity. RtW is a recognized priority by policy-makers in Finland
Benefits and harms	RtW coordination compared to usual care neither increases nor decreases the length of sick leave, and does not increase the number of workers returning to work
Certainty of the evidence^a^	Moderate quality evidence for the outcomes: Cumulative sickness absence in workdays for follow-up of 6 months and more than 12 months Proportion who had ever returned to work—long-term follow-up: 12 months Low quality evidence for the outcomes: Time to return to work: for follow-up of 6 months, 12 months and more than 12 months Cumulative sickness absence in workdays—long-term follow-up: 12 months Proportion who had ever returned to work—very long-term follow-up: more than 12 months Proportion at work at end of the follow-up—follow-up: 6 months, 12 months, more than 12 months Very low-quality evidence for the outcomes: Proportion who had ever returned to work—short-term follow-up: 6 months
Outcome importance	The included evidence does not provide information on stakeholders’ values of a possibly small decrease in the duration of sick leave. Duration of long-term sick leave in Finland lasts on average 106 days. A small decrease of sick leave by 5 days might not be that relevant to the individual worker but might be relevant for the employer, especially of small companies and blue-collar workers
Balance between desirable and undesirable effects	Coordination of RtW did not have a desirable or undesirable effect on RtW
Resource use	
Resource requirements	The analysis of the costs showed that the saving from the reduction of 5 days of sick leave outweighed by 1.6-fold the cost of RtW coordination
Certainty of the evidence	The analysis of the costs was done as a brief calculation that may not include all important items of the costs and benefits of RtW coordination, such as the costs of implementing the plan
Impact on health equity	Interventions that increase RtW improve the access to the labour market and decrease inequity between healthy and disabled workers. The effect of usual care and RtW coordination on sick leave might be similar
Acceptability	The intervention is already a common intervention by occupational health service providers in Finland [24]. We did not evaluate the attitudes of workers, employers and health service providers towards the intervention
Feasibility	Our survey showed that the intervention might not be implemented according to the recommendations. We did not evaluate important barriers that would prevent the implementation of RtW coordination in Finland

^a^The quality of evidence reflects the extent to which the review authors [6] are confident that an estimate of the effect is correct [28]

Our findings of no beneficial effect of RtW coordination is consistent with the findings of multiple other Cochrane reviews that could not find considerable effects of additional clinical interventions on RtW for workers on sick leave compared to usual care [22–26].

**Fig. 1:**
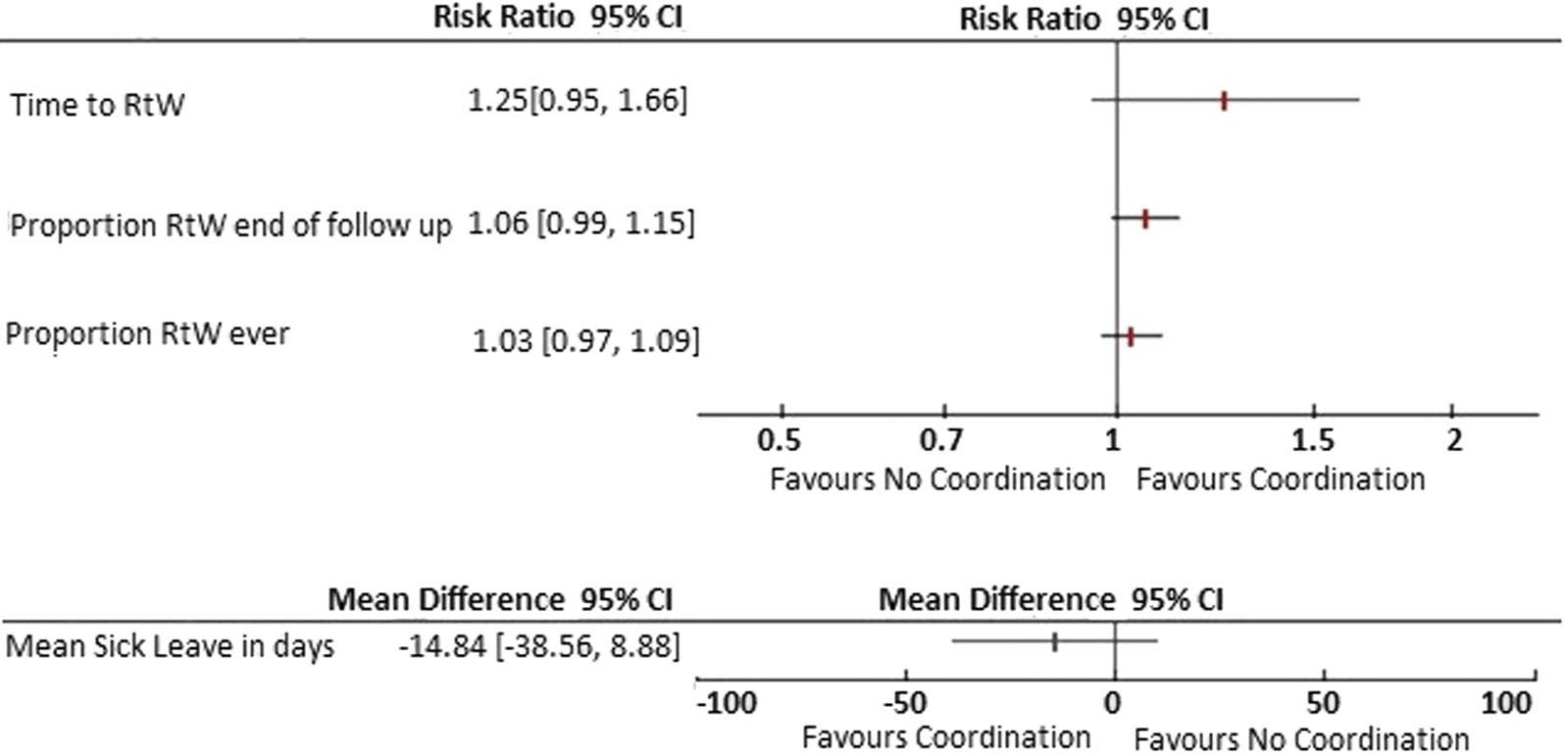
Review results: effect of RtW coordination on sick leave

The Cochrane review evaluated the effect of RtW coordination and included 14 RCTs from six European countries, Canada and the United States (Additional file 1: Table S4). The review found no beneficial effect on four outcomes across all time points, although some of the confidence intervals around the effects did not exclude a clinically relevant benefit [6]. For example, there were no statistically significant effects after 12 months on time to RtW (low-quality evidence), cumulative sickness absence (low-quality evidence), the proportion of participants at work at the end of the follow-up (low-quality evidence) or the proportion of participants who had ever returned to work (moderate-quality evidence) (Fig. 1). New eligible studies found no statistically significant effects on RtW [19, 20], but one reported a decrease in sick leave by 10 days [19–21].

Evaluation studies on the effect of RtW coordination in Finland are scarce and of less methodological quality compared to the evidence included in the Cochrane review. Results from one uncontrolled before–after study [18] and our survey of expert opinions indicated an increase in the number of workers returning to work and a decrease in the duration of sick leave compared to usual care.

The included evidence does not provide results on the importance of the outcome for participants. We judged that a small decrease in the duration of sick leave might not be relevant to the individual worker but will be for the employer, especially of small companies and blue-collar workers.

For the cost analysis, we found that RtW coordination in Finland requires on average 1.6 meetings per worker [18], with average costs of €480 per person which equals the costs of about 2 days of sick leave (Table 3). A 5-day reduction of cumulative sick leave with RtW coordination would result in €790 savings per person compared to usual care.

**Table 3:** Cost analysis of RtW coordination for an average person on >4 weeks sick leave in Finland in 2019 (Euros)

Item	Number	Costs per unit	Costs of practice as usual (PAU)	Costs of PAU plus coordination of RtW
RtW coordination meeting	1.6	300	–	480
Sick leave days	106	254	26,924	26,924
Sick leave days prevented	5	254		−1270
Total costs			26,924	26,134
Cost savings	790 (3%)			

As for acceptability, the intervention is a common intervention by occupational health service providers in Finland [24]. Further, the Ministry of Health and Social Affairs in Finland invested in RtW coordination projects [11], and the Finnish Institute of Occupational Health (FIOH) promotes and provides training courses on how to coordinate the RtW process [12].

#### Drawing conclusions

We judged RtW to be a priority for Finland due to the support of the Ministry and FIOH for RtW coordination. Joined meetings between the employer, employee and occupational health service providers have become a common RtW coordination intervention in Finland but might not be implemented well.

According to some Finnish experts in our survey and the findings from a single before–after study, RtW coordination in Finland is considered effective in increasing the number of workers returning to work and decreasing the duration of sick leave compared to no coordination of care. In contrast to these opinions, the results from the Cochrane review found no considerable desirable or undesirable effect. We found the interventions evaluated in the Cochrane review [6] to be similar to those currently implemented in Finland. New high-quality studies with large sample sizes may change the results of the review. However, research methods and findings of eligible studies published after the review were unlikely to alter the results of the review or the quality of the evidence base. The quality of the evidence of the effect of RtW coordination is of moderate to very low quality due to the imprecision of the results and the risk of bias in primary studies. We judged that the results of the review do not exclude a small beneficial effect of coordination of RtW compared to usual care.

A small reduction in the amount of sick leave days per year may not be relevant to workers from the individual perspective. From the population perspective, however, already a small reduction of 5 days of sick leave would reduce the total costs of sick leave. Our cost analysis indicated cost benefits from RtW coordination if 2 days of sick leave were averted. A possible small decrease of sick leave might result in meaningful economic consequences important for employers and society.

We judged that the resource requirements of RtW coordination were little in comparison to the costs of sick leave, but that the evidence on benefits and harms was not in favour of the intervention.

After considering all EtD framework criteria, including costs and certainty of the evidence, we conclude that investment in de-implementation strategies or better implementation of RtW coordination interventions in Finland is currently not required.

## Discussion

### Comparison with other studies

We compared the interventions included in a systematic review to the practice in one country. Previous studies described factors that influence whether a review’s conclusions can be applied to a specific setting [27, 28]. We used similar factors and prespecified criteria for our analysis, including the participants and content of the interventions.

To our knowledge, this was the first study applying the EtD framework to draw conclusions regarding (de-)implementing RtW coordination. Previous studies have shown the EtD framework a suitable tool to make policy decisions that consider the wider social and political environment. Various policy-makers and stakeholders have been involved in the development of the framework and its criteria [29], and guideline panels have used the framework for a variety of public health questions to make evidence-informed recommendations [30–34]. Further, public health decision-makers in Sweden found the EtD framework a feasible tool, even though it increased the amount of time and resources required [35].

### Strengths and limitations

We used prespecified categories (e.g. participants or content of the intervention) and judgements (identical, similar, different, unclear) to compare interventions. However, judgements remained subjective. We did not predefine what constitutes as similar (e.g. which time spans, participant characteristics), and others might judge differently. To increase the validity of the results, two authors analysed the data independently and resolved disagreements via discussion.

Routinely collected data on the quality of RtW coordination is missing, and publications on RtW interventions usually describe the ideal intervention. Empirical data on what really happens in practice are scarce. We used data from a systematic literature search including grey literature and interviewed experts in a survey to describe the (intended) content and process of the RtW coordination practice in Finland. We compared in detail the current practice in Finland to what has been evaluated in studies in the Cochrane review. We do not think that additional empirical data about Finnish practice would considerably change our conclusions about the similarities and differences between the interventions.

We combined data from different sources and study designs, but data on stakeholders’ values, attitudes, barriers to implementation of RtW coordination, and costs is either missing or very limited. Future studies that show little support from stakeholders for the intervention could alter our findings and support de-implementation strategies of RtW coordination. On the other hand, large support from stakeholders could support better implementation of RtW coordination.

Even though we do not know barriers to RtW coordination in Finland, our survey results show that coordination might not be implemented as recommended. Although our survey included a small number of participants, and results from bigger studies could alter our findings, it is questionable whether better implementation would achieve a larger decline in sick leave days. Other interventions might be better suited alternatives, such as changes in sickness certification policies or incentives for employers to improve RtW rates of their workers [34]. We do not think that additional data about implementation barriers to RtW coordination would considerably change our conclusions.

We used the EtD framework to draw conclusions on whether to (de-)implement RtW coordination in Finland. While the approach is systematic, evidence-informed and transparent, policy-makers might come to different conclusions—for example, when considering a different context in a different country or when placing different importance on certain criteria, such as costs or preferences. However, our study applied a transparent and evidence-informed approach to decision-making that has not been used previously for occupational health and safety (OHS) decisions. Therefore, our case study might be especially useful for OHS decision-makers to help ensure that all important criteria are considered and that the best available evidence is used.

## Conclusion

We found RtW coordination practice in Finland similar to the interventions and participants evaluated in the review. Consequently, the review findings apply, and the research evidence needs to be considered in decisions regarding (de-)implementing RtW coordination in Finland. Considering all EtD framework criteria, including costs and certainty of the evidence, investment in de-implementation strategies or better implementation of RtW coordination interventions in Finland is currently not required.

New studies evaluating the effect and the costs of the intervention based on better quality data would help improve the evidence base. Both would empower decision-makers to implement interventions that are clinically and economically worthwhile.

We recommend that changes in RtW practices should be implemented as part of a controlled evaluation study, including detailed descriptions of the content of interventions and usual care. New studies need to be sufficiently powerful to detect small but clinically relevant effect sizes, such as 2 days of reduced sick leave. Given the popularity of RtW coordination, an RCT of RtW coordination in Finland would be difficult to realize.

Decision-makers can use the EtD framework and its criteria as a tool to make transparent evidence-based decisions in OHS. We advise to call for a comprehensive cost–benefit analysis, an assessment of stakeholders’ values, and better-quality evidence on the effectiveness of coordination on time to RtW for Finland to better inform the decision-making process.

## Supplementary Information

**Additional file 1: Table S1**. Categories and themes for data extraction and analysis of RtW coordination interventions. **Table S2**. RtW coordination in Finland—Cost analysis factors and measures. **Table S3**. Search strategy and results April 2019. **Table S4**. Key characteristics of included trials in Vogel et al. 2017. **Table S5**. Survey results expert judgements (*n* = 10). **Table S6**. Description of usual care interventions included in the review by Vogel et al. 2017 according to main aspects of RtW coordination interventions.

## Data Availability

The datasets supporting the conclusions of this article are included within the article and its additional files.

## Abbreviations

RtW: Return-to-work
EtD: Evidence-to-decision
PAU: Practice as usual
FIOH: Finish Institute of Occupational Health
