# Supplementary material for: Evidence-informed decision about (de-)implementing return-to-work coordination to reduce sick leave: a case study
Source: Health Res Policy Syst. 2022 Feb 14;20:19. doi: 10.1186/s12961-022-00823-4 (PMC8842546; doi:10.1186/s12961-022-00823-4)
Supplement: Supplementary file 1 — Additional file 1: Table S1. Categories and themes for data extraction and analysis of RtW coordination interventions. Table S2. RtW coordination in Finland—Cost analysis factors and measures. Table S3. Search strategy and results April 2019. Table S4. Key characteristics of included trials in Vogel et al. 2017. Table S5. Survey results expert judgements (n = 10). Table S6. Description of usual care interventions included in the review by Vogel et al. 2017 according to main aspects of RtW coordination interventions. [file 12961_2022_823_MOESM1_ESM.docx]

# Additional file 1

Table S1 Categories and themes for data-extraction and analysis of RtW coordination interventions

| **Categories** | **Themes** |
| --- | --- |
| Name | key words |
| Content and process | Meetings  Workers' needs assessment  RtW plan  Implementation management RtW plan |
| Duration | Individual intervention  Including follow-up |
| Setting | Country  Place/Location  Which phase of sick leave  Which year |
| Participants | Disease of the worker  Description of sick leave  Involvement of employer (yes/no)  Provider (professions of all providers included) |
| Evaluation | Study design  Comparison  Number of participants  Outcome  Analysis method  Follow-Up  Results |

Table S2 RtW coordination in Finland - Cost analysis factors and measures

| **Factor** | **Measure** | **Notes** |
| --- | --- | --- |
| costs of sick leave | 254 Euro per day | We calculated the costs of sick leave as the average lost production including salaries (1, 2) plus personnel costs (3, 4). |
| average sick leave | 106 days per worker | We used the average total sick leave days per worker who has already been on 4 weeks sick leave based on statistical data from the Finish Social Insurance Institution (KELA) (5). |
| costs of RtW coordination | 300 Euro | We calculated the costs of RtW coordination based on our findings regarding the content of RtW coordination and usual care in Finland*. Thus, we calculated the costs of RtW coordination considering the costs of the RtW coordination meeting without adding the costs for the workers needs assessment or the implementation of the RtW plan. We used an average of 1,5 RtW coordination meetings per worker on sick leave in Finland based on 3-years registry data of a Finnish occupational health service provider (6). We estimated the costs of the meeting considering salary costs for the employer, employee, and physician (7). |
| effect of RtW coordination on sick leave | 5 days reduction per worker | We based our calculation on the effect estimate found by the Cochrane review (Analysis 1.3) (8). The meta-analysis found a statistical non-significant beneficial effect of RtW coordination after 12 months follow up but showed substantial heterogeneity in studies. In our model we used an effect of RtW coordination that was not excluded by the analysis and assumed a reduction of 5 days of annual sick leave per worker on long term sick leave. |
| **RtW:** return to work; ***** Content of RtW interventions in Finland: a) a basic workers needs’ assessment is part of RtW coordination and usual care), b) a RtW plan is developed during a RtW coordination meeting and can be directly implemented, c) the RtW coordination meeting is additional to care as usual, and d) changes to the plan and evaluation of the implementation are dealt with in follow-up meetings | | |
| **References:**  **1)** Rissanen M, Kaseva E. Menetetyn työpanoksen kustannus. Ministry of Social Affairs and Health, Sosiaali-ja terveysministeriön työsuojeluosasto, strateginen suunnitteluryhmä; 2014.  **2)** Statistics Finland. Index of wage and salary earnings. https://www.stat.fi/til/ati/tau_en.html. Accessed 15. May 2019. **3)** Verohallinto. Työnantajan ja työntekijän vuoden 2019 sosiaalivakuutusmaksuprosentit. Updated 28.12.2018. https://www.vero.fi/tietoa-verohallinnosta/verohallinnon_esittely/uutiset/uutiset/2018/työnantajan-ja-työntekijän-eläke--ja-vakuutusmaksuprosentit-2019/. Accessed 16. May 2019. **4)** Ilmarinnen. Employer’s social insurance contributions and income limits in 2019. https://www.ilmarinen.fi/en/employer/employers-social-insurance/. Accessed 16. May 2019. **5)** KELA. Sairauspäivärahojen saajat ja maksetut sairauspäivärahat 2017. http://raportit.kela.fi/ibi_apps/WFServlet?IBIF_ex=NIT098AL. Accessed 15. May 2019. **6)** Reho T, Atkins S, Talola N, Ojajärvi U, Sumanen M, Viljamaa M, et al. Työterveysneuvottelut työssä jatkamisen tukena – kuvaileva tutkimus. Suomen Lääkärilehti. 2018;73(36):1948-53. **7)** Harju A. Kannattavatko työterveysneuvottelut?. https://research.uta.fi/ttavain/kannattavatko-tyoterveysneuvottelut/. Accessed 16. May 2019. **8)** Vogel N, Schandelmaier S, Zumbrunn T, Ebrahim S, de Boer WE, Busse JW, et al. Return-to-work coordination programmes for improving return to work in workers on sick leave. Cochrane Database of Systematic Reviews. 2017;3:Cd011618. | | |

Table S3 Search strategy and results April 2019

| **Cochrane Library, run 18.03.2019** | | |  |
| --- | --- | --- | --- |
| **search** | **Query** | **results** | **included** |
| #1 | (Coordination AND return to work [in Title Abstract Keyword]) AND Vogel [in Author] | 1 | 1 |
| **PubMed, last run 26.04.2019** | | |  |
| **search** | **Query** | **results** | **included** |
| #1 | Finnish OR Finland | 160023 | - |
| #2 | "occupational health" | 74442 | - |
| #3 | workability OR work-ability OR "work ability" OR "sickness absence" OR sick-leave OR "sick leave" OR sickleave OR "return to work" | 18503 | - |
| #4 | cooperation OR collaboration OR meeting* OR negotiation* OR coordination | 401625 | - |
| #5 | #1 AND #2 AND #3 AND #4 | 17 | 2 |
| **Webpages and snowball principle, last search 29.04.2019** | | |  |
| **webpage** | **provider** | **results** | **included** |
| [www.uta.fi](http://www.uta.fi) | University of Tampere | 33 | 4 |
| [www.ttl.fi](http://www.ttl.fi) | Finnish Institute of Occupational Health |  |  |
| [www.thl.fi](http://www.thl.fi) | National Institute for Health and Welfare |  |  |
| <https://stm.fi> | Ministry of Social Affairs and Health |  |  |

Table S4 Key characteristics of included trials in Vogel et. al 2017

| **14 RCTs that compared return-to-work coordination programmes to usual practice (data from Vogel et. al 2017)** | | | | | | |
| --- | --- | --- | --- | --- | --- | --- |
| **Reference ID*** | **Control intervention^1^** | **Country** | **Intervention year(s)** | **Reason for workers sick leave** | **RtW Outcome(s)** |  |
| Bültmann 2009 | moderate level of support | Denmark | 2004-2005 | low back pain or musculoskeletal disorders as the main cause of sick leave | cumulative sickness absence, proportion of participants at work at end of the follow-up |  |
| Davey 1994 | low level of support | Scotland, North-East England | unknown | injuries likely to result in absence from work of 6 months or more | proportion of participants at work at end of the follow-up, proportion of participants who had ever returned to work |  |
| Donceel 1999 | low level of support | Belgium | 1996-1998 | surgery for disc herniation | time to return to work, proportion of participants at work at end of the follow-up, proportion of participants who had ever returned to work |  |
| Feuertsein 2003 | moderate level of support | USA | 1999-2002 | work-related upper extremity disorder | time to return to work, proportion of participants who had ever returned to work |  |
| Jensen 2012 | high level of support | Denmark | 2004-2009 | low back pain | cumulative sickness absence, time to return to work, proportion of participants at work at end of the follow-up, proportion of participants who had ever returned to work |  |
| Lambeek 2010 | moderate level of support | Netherlands | 2005-2008 | non-speciﬁc chronic low back pain | cumulative sickness absence, time to return to work, proportion of participants who had ever returned to work |  |
| Lindh 1997 | low level of support | Sweden | not reported | non-speciﬁc chronic musculoskeletal pain | proportion of participants at work at end of the follow-up, proportion of participants who had ever returned to work |  |
| Myhre 2014 | moderate level of support | Norway | 2009-2012 | neck pain (10%) and low back pain (90%) | cumulative sickness absence, time to return to work, proportion of participants who had ever returned to work |  |
| Purdon 2006 | low level of support | United Kingdom | 2003-2005 | any condition likely to result in a 50% chance to return to work (musculoskeletal, mental and behavioural problems, injuries) | proportion of participants at work at end of the follow-up, proportion of participants who had ever returned to work |  |
| Rossignol 2000 | low level of support | Canada | 1995-1997 | any work-related injury to the middle or lower vertebral column, not surgery or multiple injuries | time to return to work, proportion of participants who had ever returned to work |  |
| Scholz 2015 | moderate level of support | Switzerland | 2002-2012 | severe accidents, occupational and non-occupational | cumulative sickness absence |  |
| Stapelfeldt 2011 | high level of support | Denmark | 2007-2009 | low back pain | cumulative sickness absence, time to return to work, proportion of participants who had ever returned to work |  |
| Van der Feltz Cornelis 2010 | moderate level of support | Netherlands | not reported (3 years) | anxiety, depression, somatoform disorder | time to return to work, proportion of participants at work at end of the follow-up, proportion of participants who had ever returned to work |  |
| Volker 2015 | moderate level of support | Netherlands | not reported | common mental disorders | cumulative sickness absence, time to return to work, proportion of participants who had ever returned to work |  |
| ^1^ defined by Vogel et al. 2017 as usual practice with low, moderate or high level of support | | | | | | |
| *Citations to reference IDs: **Bültmann** U, Sherson D, Olsen J, Hansen CL, Lund T, Kilsgaard J. Coordinated and tailored work rehabilitation: a randomized controlled trial with economic evaluation undertaken with workers on sick leave due to musculoskeletal disorders. Journal of Occupational Rehabilitation 2009;19(1):81–93.; **Davey** CA. The Implementation and Evaluation of a Rehabilitation Co-ordinator Service for Personal Injury Claimants. Edinburgh: University of Edinburgh, 1994. **Donceel** P, Du Bois M, Lahaye D. Return to work after surgery for lumbar disc herniation. A rehabilitation-oriented approach in insurance medicine. Spine 1999;24(9):872–6.; **Feuerstein** M, Huang GD, Ortiz JM, Shaw WS, Miller VI, Wood PM. Integrated case management for work-related upper-extremity disorders: impact of patient satisfaction on health and work status. Journal of Occupational and Environmental Medicine 2003;45(8):803–12.; **Jensen** C, Jensen OK, Nielsen CV. Sustainability of return to work in sick-listed employees with low-back pain. Two-year follow-up in a randomized clinical trial comparing multidisciplinary and brief intervention. BMC Musculoskeletal Disorders 2012;13:156.; **Lambeek** LC, van Mechelen W, Knol DL, Loisel P, Anema JR. Randomised controlled trial of integrated care to reduce disability from chronic low back pain in working and private life. BMJ 2010;340:c1035. [DOI: 10.1136/bmj.c1035; **Lindh** M, Lurie M, Sann H. A randomized prospective study of vocational outcome in rehabilitation of patients with non-speciﬁc musculoskeletal pain: A multidisciplinary approach to patients identiﬁed after 90 days of sick-leave. Scandinavian Journal of Rehabilitation Medicine 1997;29(2): 103–12.; **Myhre** K, Marchand GH, Leivseth G, Keller A, Bautz-Holter E, Sandvik L, et al. The effect of work-focused rehabilitation among patients with neck and back pain: a randomized controlled trial. Spine 2014;39(24):1999–2006.; **Purdon** S, Stratford N, Taylor R, Natarajan L, Bell S, Wittenburg D. Impacts of the job retention and rehabilitation pilot. Leeds: Department for Work and Pensions. 2006. Research Report No 342.; **Rossignol** M, Abenhaim L, Seguin P, Neveu A, Collet JP, Ducruet T, et al. Coordination of primary health care for back pain. A randomized controlled trial. Spine 2000;25 (2):251-8; discussion 258-9.; **Scholz** SM, Andermatt P, Tobler BL, Spinnler D. Work incapacity and treatment costs after severe accidents: standard vs. intensive case management in a 6-year randomized controlled trial. Journal of Occupational Rehabilitation 2016;26(3):319–31.; **Stapelfeldt** CM, Christiansen DH, Jensen OK, Nielsen CV, Petersen KD, Jensen C. Subgroup analyses on return to work in sick-listed employees with low back pain in a randomised trial comparing brief and multidisciplinary intervention. BMC Musculoskeletal Disorders 2011;12:112.; **Van der Feltz-Cornelis** CM, Hoedeman R, de Jong FJ, Meeuwissen JA, Drewes HW, van der Laan NC, et al. Faster return to work after psychiatric consultation for sicklisted employees with common mental disorders compared to care as usual. A randomized clinical trial. Journal of Neuropsychiatric Disease and Treatment 2010;6:375–85.; **Volker** D, Zijlstra-Vlasveld MC, Anema JR, Beekman AT, Brouwers EP, Emons WH, et al. Effectiveness of a blended web-based intervention on return to work for sick-listed employees with common mental disorders: results of a cluster randomized controlled trial. Journal of Medical Internet Research 2015;17(5):e116. | | | | | | |

Table S5 Survey results expert judgements (n=10)

|  | Survey ^a^ (n=6) | Survey ^b^ (n=4) | **Percentage of all respondents (n=10)** |  |
| --- | --- | --- | --- | --- |
| **Over the last three years how many workers in Finland, that have been on sick leave for more than 4 weeks, received occupational health negotiations (“työterveysneuvottelu”)?** | | | | |
| more than 80% | 0 | 0 | **0%** |  |
| at least 50% but less than 80% | 3 | 0 | **30%** |  |
| less than 50% but at least 20% | 1 | 1 | **20%** |  |
| less than 20% | 2 | 3 | **50%** |  |
| Comments | 1 | 0 |  |  |
| Comment 1: Most of the sick leaves of this length end without a need for occupational health negotiation | | | | |
| **What is the difference between the process of occupational health negotiations most commonly conducted in Finland during the last three years (below referred to as usual practice) and the process now described on the e-learning platform "Työterveysneuvottelu-ratkaisuja-työhön” (below referred to as best practice)?** | | | | |
| Best practice does include an assessment of the worker’s needs, usual negotiation practice does not. | 3 | 2 | **50%** |  |
| Best practice does lead to an individually tailored return-to-work plan directed at the worker, the workplace, and the employer that includes more than one possible action (e.g. treatment and work accommodation), usual negotiation practice does not. | 4 | 2 | **60%** |  |
| With best practice the affected worker has at least one joint face-to-face meeting with the occupational physician and the employer/supervisor (and maybe others such as occupational health nurse), in usual negotiation practice the worker has no joint face-to-face meetings with the occupational physician and the employer/supervisor. | 1 | 1 | **20%** |  |
| There is no difference between best practice and usual practice for occupational health negotiations in Finland. | 0 | 1 | **10%** |  |
| Other (please specify): | 0 | 0 |  |  |
| **What is/are the main difference/s between occupational health negotiations and other return-to-work interventions (usual care) in Finland?** | | | | |
| Compared to occupational health negotiations, in most cases, other interventions only focus on treatment and health or work capacity evaluations and an assessment of possible work accommodations is missing. | 2 | 3 | **50%** |  |
| In most cases, with other interventions the worker receives no guidance on how to return-to-work, but with occupational health negotiations he does. | 3 | 2 | **50%** |  |
| In most cases, with other interventions the advice on how to return-to-work is only general but with occupational health negotiations the worker receives an individually tailored return-to-work plan directed at the worker, the workplace, and the employer. | 4 | 1 | **50%** |  |
| In most cases, other interventions do not include joined face-to-face meeting(s) with occupational health services and workplace representative, but occupational health negotiations do. | 3 | 3 | **60%** |  |
| None of the above. | 0 | 0 | **0%** |  |
| Other (please specify): | 0 | 0 | **0%** |  |
| **Which intervention(s) are more effective?** | | | | |
| All interventions are similar effective in the length of sick leave and the number of workers returning to work | 1 | 1 | **20%** |  |
| Occupational health negotiations are more effective than other interventions (worker take shorter sick-leaves and/or more worker return-to-work) | 1 | 4 | **50%** |  |
| Occupational health negotiations that fulfil all criteria advocated on the e-learning platform are more effective than occupational health negotiations that don't fulfil all criteria (worker take shorter sick-leaves and/or more people return-to-work) | 3 | 1 | **40%** |  |
| Other outcome or statement (please specify) (comments) | 2 | 0 | **20%** |  |
| Comment 1: depending on the circumstances (original: “valitaan tilanteen mukaan”)  Comment 2: Effectiveness of the interventions need to be assessed in their own contexts (based on the case in question). It is impossible to compare effectiveness of the different interventions directly. The model provided for ideal intervention on the e-learning platform does not result to shorter sick-leaves and/or more people return-to-work but suggest a smoothier negotiation process in the occupational health negotiation and joint decision making concerning RtW. | | | | |
| **Total number of respondents:** | **6** | **4** |  |  |
| ^a^ researcher ^b^ training course participants | | | | |

Table S6 Description of usual care interventions included in the review by Vogel et al. 2017 according to main aspects of RtW coordination interventions

|  | **Frequency** | **Participants** | **Setting** | **Type (type of contact)** | **Content** |
| --- | --- | --- | --- | --- | --- |
| **Contact** | low level support: no restrictions | low level support: no restrictions  moderate level support: contact to occupational physician, general practitioner and mental health professional, contact to other healthcare professionals not restricted | low level support: no restrictions | low level support: no restrictions  high level support: coordination between stakeholders  moderate level support:  multidisciplinary intervention, contact or no personal contact | low level support: no restrictions,  moderate level support:  Medical records and return-to-work schedules were sent to participants and their general physician |
| **workers’ needs assessment** | low level support: no restrictions, | low level support: no restrictions,  high level of support  by a physician | low level support: no restrictions, | low level support: no restrictions  high level support and moderate level support:  standard low back pain or clinical examination | low level support: no restrictions,  moderate level of support:  relevant imaging, information about the ﬁndings,  emphasis on removing fear-avoidance beliefs, restoring activity level, and enhancing  self-care and coping"  ensure that the patient receives the rehabilitation deemed necessary  focus on medical care  high level of support: clinical examination, relevant imaging and examinations ordered, and treatment options discussed |
| **RtW plan** | low level support: no restrictions | low level support: no restrictions | low level support: no restrictions | low level support: no restrictions  high level support: advice | low level support: no restrictions,  high level of support: to resume work when possible, adjusted medical pain management, physiotherapy  moderate level of support:  ensure that the patient receives the rehabilitation deemed necessary  focus on medical care |
| **Implementation management** | low level support: no restrictions | low level support: no restrictions | low level support: no restrictions | low level support: no restrictions  moderate level of support: standard management procedure | low level support: no restrictions  moderate level of support: focus on handling acute emerging problems and helping with return to work |
